# Estimating Braking Performance in Osteoarthritis of the Knee or Hip with a Reaction Timer

**DOI:** 10.1111/os.12446

**Published:** 2019-04-03

**Authors:** Maximilian von Bernstorff, Jennifer Rapp, Felix Bausenhart, Martina Feierabend, Ingmar Ipach, Ulf K Hofmann

**Affiliations:** ^1^ Department of Orthopaedic Surgery Medical Faculty of the University of Tübingen Tübingen Germany; ^2^ Department of Orthopaedic Surgery University Hospital of Tübingen Tübingen Germany; ^3^ Department of Orthopaedic Surgery, Division of Neuropsychology Hertie Institute for Clinical Brain Research Tübingen Germany; ^4^ Department of Orthopaedic Surgery MVZ Orthopädie Straubing Straubing Germany

**Keywords:** Automobile driving, Brake response time, Hip arthroplasty, Knee arthroplasty, Osteoarthritis

## Abstract

**Objective:**

To investigate if testing in a brake simulator can be replaced by a simple reaction timer setup imitating the ergonomic conditions of emergency braking when evaluating the ability to drive in patients with musculoskeletal problems of the lower extremities.

**Methods:**

A cross‐sectional survey was performed in the Department of Orthopaedic Surgery in our University Hospital from October 2014 until May 2015. Patients attending our department with either osteoarthritis or arthroplasty of the knee or hip were asked to participate in the study if they had a valid driving license. The age limit was from 18 to 85 years. Both women and men were included. Registered demographic data were patient age, height, sex, body weight, and body mass index. Braking performance (brake response time [BRT]) was evaluated in a brake simulator that was embedded into a real car cabin (10 measurements). The values obtained were compared with those registered when simply testing (5 measurements) those patients with a normal reaction timer setup that imitated the sitting position in a car. Kendall's tau correlation coefficient was calculated between the values obtained from the brake simulator with those from the reaction timer setup.

**Results:**

Altogether, 137 patients (median age 67 years [range, 24–89 years]) with either osteoarthritis of the knee (n = 55) or hip (n = 82) were tested. Age was comparable in both collectives (*P* = 0.807). The mean body height was 1.70 m in both groups. Knee patients presented with a higher body weight of approximately 5 kg (*P* = 0.014) and consequently also had a higher body mass index (*P* = 0.023). The median BRT in the brake simulator was 628 ms (range, 390–1444 ms) for all subjects: 592 ms (range, 418–1146 ms) in the hip group and 696 ms (range, 390–1444 ms) in the knee group. Measurement values obtained by the reaction timer were significantly (*P* < 0.001) higher by approximately 15% (SD, 22%) than those measured in the brake simulator. A moderate correlation was found between the reaction timer and the brake simulator, with a Kendall's tau of 0.449 (*P* < 0.001) for all patients. Interestingly, hip patients showed a higher correlation (*τ* = 0.471) than knee patients (*τ* = 0.263).

**Conclusion:**

Even though the measured correlations do not allow us to make a definite statement concerning braking performance, especially in knee patients, a simple reaction timer test can provide a low‐cost first estimate of BRT for patients and their treating physicians. For forensic statements, the brake simulator will, however, remain the gold standard.

## Introduction

Individual mobility, which in our modern society is largely ensured by the use of a motor car, plays a crucial role in social participation and quality of life. Beginning in the second half of the 20th century, the total amount of yearly driven kilometers has been continually rising in the United States^1^, ^2^. With an aging population, the number of elderly drivers increases as well. It has been predicted that by 2020, drivers aged 65 and older will represent over 16% of the driving population in the United States^3^. One key element of safe driving is the ability to perform effective emergency braking. To this end, a certain degree of fitness and vigilance is required.

Hence, numerous studies focus on the ability of patients with musculoskeletal disorders of the lower extremities to perform an emergency stop. Generally, these studies investigate the required time after surgery to be able to drive again, thereby mostly taking as the baseline preoperative values that were already impaired. From these studies, some general recommendations have been formulated: For both right‐side total knee and right‐side total hip arthroplasty, it has been suggested that patients abstain from driving for 4–8 weeks after surgery^4^, ^5^, ^6^, ^7^. After arthroplasty on the left side, a 2‐week interval has been proposed^8^. Patients with osteoarthritis (OA), however, also show impaired braking performance compared with an age‐matched control group^9^.

The problem with all these recommendations is the strong interindividual variability, which makes it impossible to predict individual brake response time (BRT) based simply on time after surgery or OA findings on radiographs. While Jordan *et al*. (2014) suggest abstaining from driving for 6 weeks after total hip arthroplasty, they also state that individual examinations and recommendations are necessary, which in the end means individual testing in a brake simulator^7^. Even though in their study the overall BRT dropped continually after surgery, the distribution of the individual median BRT of 10 performed measurements per subject had a relevant standard deviation of 129 ms around the mean value and a range of approximately 400 ms for patients with right total hip arthroplasty at 6 weeks after surgery. This means that 35% of these patients were still above the 600 ms threshold recommended for this experimental setup^9^. Hence, individual testing in a brake simulator is necessary to provide an individualized statement about driving aptness. As such simulators are not generally available, an easier alternative is needed. Some studies have already correlated BRT with various clinical tests or surveys to find potential alternatives to a simulator. No correlation was observed with the Kellgren–Lawrence grade for OA (Kendall's tau: *τ* = 0.007; significance level: *P* = 0.92)^10^, but Hau *et al*. (2000) described a positive correlation between BRT and their step and standing tests for patients after knee arthroscopy (step test: *r* = −0.45/−0.79; standing test: *r* = −0.35/−0.70; both *P* < 0.001)^11^. None of these tests allow, however, a forensically valid prediction of braking performance, which makes the cumbersome brake simulator measurements a persistent necessity.

The aim of the present study was to evaluate braking performance by means of a simple and cheap reaction timer with a setting that is similar to driving a car and to compare these braking times with those obtained in a brake simulator in a real car cabin.

## Materials and Methods

### *Inclusion and Exclusion Criteria*

Patients attending our department were asked at random to participate in the study.

Inclusion criteria were: (i) possession of a valid driving license; (ii) patient age between 18 and 85 years; and (iii) OA of the right or left hip/knee or hip/knee replacement.

Exclusion criteria were: (i) use of a walking frame; (ii) cardiac insufficiency (NYHA 3–4); (iii) a recent heart attack or stroke within the previous 6 months; (iv) recent fractures; (v) systemic or metastasized cancer; (vi) a peripheral sensorimotor deficit with a grade of <3/5 on the Medical Research Scale for muscle strength; and (vii) drug intake known to affect reaction time**.**

### *Patients*

Registered demographic data were patient age, height, sex, body weight, and body mass index.

Patients were tested during consultation or before a planned hip or knee replacement in our department in this cross‐sectional study, which is part of a larger investigation of BRT (Clinicaltrials.gov: Identifiers: NCT02175160 and NCT02308813 Unique Protocol ID: 619/2013BO2 and 503/2014BO2). The recruitment time was from October 2014 to May 2015. Written informed consent was received from all patients before participation. Full institutional, departmental, and local ethical committee approvals were obtained before commencement of the study (project numbers of the ethics committee of the University of Tübingen: 619/2013BO2; 503/2014BO2).

### *Testing in the Brake Simulator*

**Figure 1:**
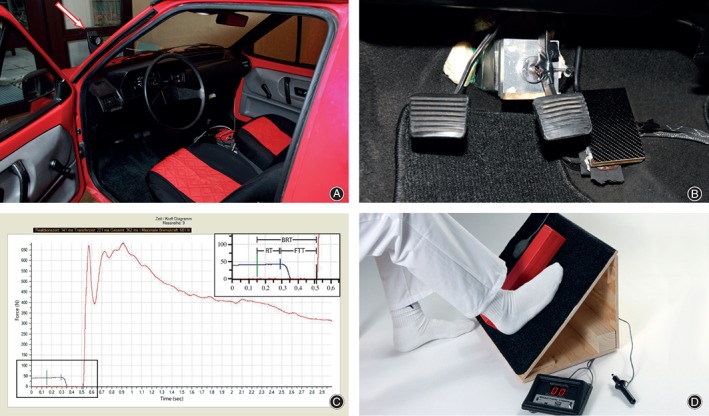
Brake simulator and recorded data: (A) Brake simulator incorporated in a Volkswagen Polo 2 to measure brake response time (BRT), with its components reaction time (RT) and foot transfer time (FTT), the arrow indicating the red LED that represents the signal for emergency braking. (B) Brake pedal in the middle and accelerator on the right, both equipped with a pressure sensor. On the left is the clutch pedal. (C) Measurement graph generated by the software, showing reaction time, foot transfer time, and brake response time. The top right insert is an enlargement of the bottom left section of the graph. At the beginning of the left graph the black horizontal line displays the pressure on the accelerator. The vertical green line marks the triggering of the red emergency signal. Reaction time is measured between the green and vertical blue line, which highlights the beginning of pressure decrease on the accelerator pedal. This is also the starting point for foot transfer time. No pressure is measured while the foot is transferred to the brake pedal. The endpoint for foot transfer time is the vertical black line on the right, which represents the beginning of pressure increase on the brake pedal, shown in the red graph. (D) Experimental setup with a simple reaction timer. Upon flashing of a red LED the foot is to be lifted over the red block on an inclined plane to push a detector pad placed on the other side of the block to register brake response time^9^.

As the baseline reference for braking performance, we used the same experimental setup as described previously^9^. The measurement equipment was incorporated in a Volkswagen Polo 2 automobile to allow measurements under realistic ergonomic conditions (Fig. 1A).

Pushing the accelerator continually started the registration process. Within a random interval of 10 s, the supervisor activated a red light‐emitting diode (LED) placed in front of the windscreen at the driver's eye level. Participants were instructed to consider this flashing light as the emergency signal upon which they should perform an emergency braking process. After three test trials, this procedure was repeated 10 times.

Both the accelerator and brake pedal were equipped with force transducers, which were connected to a measurement amplifier to send their signals to a registration module (Fig. 1B). The LED was likewise connected to this module, from which the information was sent to the processing computer equipped with a custom‐made software program (Fig. 1C). This setup allowed us to measure both reaction time and foot transfer time, which together form the BRT, which has been frequently used in the literature to evaluate braking performance. Reaction time was thereby defined as the time elapsed between flashing of the LED and the beginning of pressure decrease on the accelerator.

For the experimental setup used in the present study, earlier studies found no significant learning effect for reaction time, foot transfer time, or BRT^9^.

### *Testing with the Reaction Timer*

The reaction timer test was created in such a way that it has a similar ergonomic setup as that in a motor vehicle. A chair with adjustable height allowed patients to sit with both hips and knees flexed at 90°. A 45° inclined plane was placed at a distance so that the patients’ right foot could be placed on it with the knee in 45° flexion. At the center of this inclined plane, a red cuboid, 5 cm in width and height, was fixed along the longitudinal axis (Fig. 1D). The detection pad of a reaction timer (American Educational Products, Fort Collins, CO, USA) was then placed on the inclined plane on the left side of the red cube. With a hand trigger mechanism out of sight of the patient, the supervisor activated an integrated red LED placed at the patients’ eye level, which simultaneously started the registration process. In accordance with measurements made in the brake simulator, patients were asked to consider this red light as the emergency signal upon which to perform “emergency braking” in the sense that they should lift their right foot placed on the inclined plane on the right side of the cube and transfer it to the other side of the cube as quickly as possible to touch the pad and, thus, end the measurement. After three practice trials, this test was performed five times and, thus, the necessary time measured to successfully respond to the signal. The maximum time elapsed detectable by the device is 1 s. Patients with measurements exceeding this value were excluded from the study analyses.

### *Statistical Analysis*

Patients with missing data were excluded from the study. Distribution of variables was judged by histograms. Data are reported as means (standard deviation) or median (minimum–maximum) as appropriate. All patients were analyzed as a joint group and then a comparative analysis was performed between patients with hip and patients with knee pathology. Differences between the hip and the knee group were evaluated by *t*‐test for independent samples, Mann–Whitney *U*‐test, and χ^2^‐test, as appropriate. Comparison between BRT and reaction timer testing was performed using the Wilcoxon test. All inference tests were performed with a two‐tailed significance level of *P* = 0.05 without adjusting for multiple testing. Correlations between the BRT from the brake simulator and the time measured in the reaction timer setup were carried out by Kendall's tau rank correlation and data are presented in the form of scatterplots and boxplots. Statistical evaluation was performed by using IBM SPSS 21 (IBM Corporation, Armonk, NY, USA).

## Results

### *The Study Collective*

Initially, 160 patients were included in the study, of whom 4 with knee OA were unable to complete the testing procedure. For 19 patients, the reaction timer measured an elapsed time of 1 s and they were, therefore, also excluded from the analyses. In total, 82 patients with hip pathology (OA: 49 and arthroplasty: 33) and 55 with knee pathology (OA: 39 and arthroplasty: 16) were analyzed. The median age was 67 years (range, 24–89 years), with no significant difference between the knee and hip patients (*P* = 0.807). Although more women (n = 74) were analyzed than men (n = 63), the distribution of women and men among the subgroups was not significantly different. The mean body height was 1.70 m in both groups. Knee patients presented with a higher body weight of approximately 5 kg (*P* = 0.014) and, consequently, also had a higher body mass index (*P* = 0.023) (Table 1).

**Table 1:** Demographic data

Parameter	Study group	Hip group	Knee group	
	(*n* = 137)	(*n* = 82)	(*n* = 55)	*P*‐value
Age (years)*	67 (24–89)	67 (24–84)	68 (31–89)	0.807†
Men (cases)	63	42	21	0.133‡
Women (cases)	74	40	34	
Body height (m)§	1.70 (0.09)	1.70 (0.10)	1.70 (0.08)	0.817**
Body weight (kg)*	80 (43–145)	78 (43–123)	83 (52–145)	0.014† ^,^ ††
Body mass index (kg/m^2^)*	28 (19–47)	27 (19–44)	29 (20–47)	0.023† ^,^ ††

Demographic characteristics presented as median (minimum–maximum)* or mean (standard deviation)^§^ as appropriate.

‡ Pearson χ^2^‐test

† Mann–Whitney *U*‐test, and

** *t*‐test for independent samples to compare the hip and knee group composition

†† Significant *P*‐values.

### *Braking Performance in the Simulator and with the Reaction Timer*

**Figure 2:**
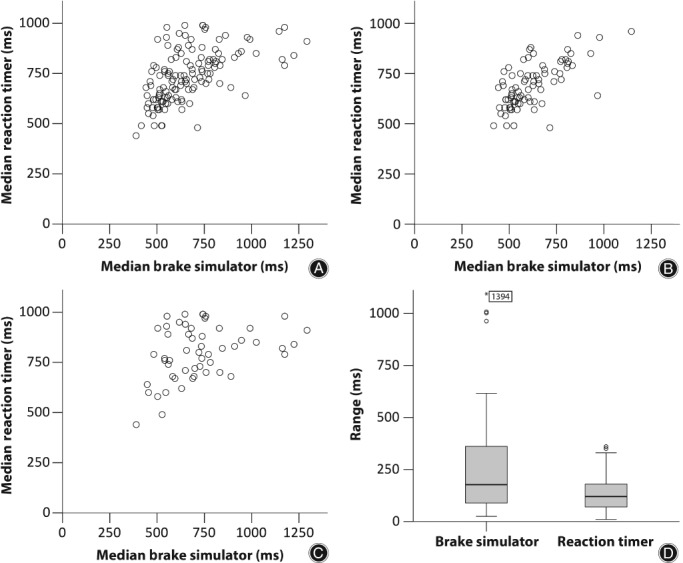
Scatterplots of correlations between median brake response time (BRT) and median time with the reaction timer for (A) all subjects (*n* = 137), (B) the hip cohort (*n* = 82), and (C) the knee cohort (*n* = 55). (D) Showing the distribution of individual ranges (minimum–maximum) over all measurements with the brake simulator and with the reaction timer. For better comparability with the reaction timer only 5 random measurements of the 10 performed in the brake simulator were selected.

The median BRT in the brake simulator was 628 ms (range, 390–1444 ms) for all subjects: 592 ms (range, 418–1146 ms) in the hip group and 696 ms (range, 390–1444 ms) in the knee group. The values obtained using the reaction timer setup were all slightly higher by a mean of 15% (SD = 22%) than in the brake simulator (*P* < 0.001), with a median time of 730 ms (range, 440–990 ms) for all subjects, of 690 ms (range, 460–960 ms) for hip patients, and of 810 ms (range, 440–990 ms) for knee patients. A moderate correlation was found between the reaction timer and the brake simulator, with a Kendall's tau of 0.449 for all patients and an even higher correlation of *τ* = 0.471 for hip patients. In contrast, knee patients presented with only a fair correlation of *τ* = 0.263 (Table 2). This result can also be seen in the distribution of the measurement pairs in the form of scatterplots, where the distribution of points is condensed in the hip group and only loosely arranged in the knee group (Fig. 2A–C).

**Table 2:** Braking performance in the brake simulator and with the reaction timer setup

Parameter	Study group (*n* = 137)	Hip group (*n* = 82)	Knee group (*n* = 55)
Braking performance with the reaction timer (ms)*	730 (440–990)	690 (460–960)	810 (440–990)
Brake response time with brake simulator (ms)	628 (390–1444)	592 (418–1146)	696 (390–1444)
Kendall's tau (τ)	*τ* = 0.449, *P* < 0.001	*τ* = 0.471, *P* < 0.001	*τ* = 0.263, *P* < 0.005

* Value output by the reaction timer is rounded to the hundredth of a second. Values are presented as the median (minimum‐maximum).

### *Wide Range of Data Distribution in Both of the Two Testing Methods*

It is important to realize, however, that a substantial range can be observed between the best and worst values in both experiments: While the median value for the 10 measurements is 177 ms in the brake simulator, the lowest value is 25 ms and the highest 1394 ms. For the measurements with the reaction timer, the median is 120 ms, with a minimum of 10 ms and a maximum of 360 ms (Fig. 2D).

## Discussion

The aim of this study was to evaluate whether a simple testing method such as a reaction timer can be used to adequately estimate the braking performance of patients with OA or arthroplasty of the knee or hip. To this end, we tested 137 patients in a brake simulator and compared the BRT results with the times obtained from an ergonomically similar setup using a reaction timer and simulating the braking movement of the right foot across a cuboid attached to an inclined plane. The observed BRT for the different patient groups are in line with those reported in previous studies of hip and knee patients in our department^4^, ^9^.

### *Interpretation of Results*

The results of the brake simulator were approximately 100 ms faster than those from the reaction timer experiment for both hip and knee patients. Both emergency signals came from a red LED and the required movement of the right leg was comparable. The difference in time can, therefore, probably be attributed to the lack of a steering wheel to hold on to, which requires additional muscle activation to stabilize the trunk for this movement.

Direct comparison of the results from both types of measurement shows a significant (*P* < 0.001), fair^12^ correlation by Kendall's tau of 0.449. The correlation for hip patients was higher at *τ* = 0.471, *P* < 0.001, than it was for knee patients at *τ* = 0.263, *P* < 0.005. We can only speculate about the reasons for this notable difference. Patients with hip problems did, however, perform better in both experiments, a finding that is consistent with results from previous studies^4^, ^7^, ^9^. One possible reason for this observation is that for foot transfer from the accelerator to the brake pedal, the knee joint is more involved than the hip joint, which makes the impact of pathologic conditions in knee joints on, for example, arthrogenic muscle inhibition^13^, ^14^, ^15^, more relevant.

Although a direct comparison of the range between the two tests performed is not possible because only five measurements were performed with the reaction timer and the upper registration limit is 1 s, the wide range of the results for a single patient in both tests is noteworthy. The median individual range was 120 ms for the reaction timer and 177 ms for the brake simulator, with the maximum values reaching a range of well beyond 1 s. This high measurement variability could also explain why an even better overall correlation could not be observed between these testing methods.

### *Contextualization*

In terms of the practical impact of this finding, of note is that the observed variabilities were obtained under standardized testing conditions with an expected event with no cognitive load and no decision‐making process required to initiate the pre‐programmed motor response. It is likely that under real traffic conditions, variability is even stronger, as BRT increases as a function of expectancy and cognitive load^16^, ^17^.

Several investigators have attempted to find a simple clinical test or measurement protocol to replace the tedious brake simulator measurements^11^, ^18^, ^19^, ^20^, ^21^, ^22^ with, however, few encouraging results. To the best of our knowledge, no study has thus far compared results obtained in a brake simulator with an easily implementable reaction timer setup that imitates the brake simulator. Because the reaction timer experiment is as close to a brake simulator as possible in comparison to other conceivable clinical tests, we believe that the correlation observed is as good a result as we can expect when trying to replace the brake simulator.

Nonetheless, the question of whether brake simulators can be replaced by such setups in everyday practice can only be answered ambiguously. For research purposes, it should be self‐evident that the best simulator design should be used to provide gold‐standard values. For the everyday routine, however, this problem can be solved only by finding an answer to a different question: To what extent do brake simulator results reflect actual emergency braking performance in real traffic? Although this issue will be difficult to address, it would be essential to know whether a brake simulator leads to more reliable results than, for example, a simple reaction timer experiment. Given the wide range of results observed in both brake simulator and reaction timer experiments, we believe that such a simple setup ought to be sufficient for providing a first estimate of braking performance. While a maximum BRT of 600 ms was established for the brake simulator used in this study^9^, the values obtained for the reaction timer were approximately 100 ms higher, so that we would argue for a maximum time limit under these conditions of approximately 700 ms.

### *Study Limitations*

The upper time limit of 1 s for the reaction timer forbids a direct comparison between ranges of brake simulator and reaction timer measurements. It also leads to bias in the sense that measurements with values over 1 s in the reaction timer experiment could not be included. Because the idea of the reaction timer experiment is to allow a first impression of braking performance, however, results exceeding 1 s are so far beyond any recommended reference values that this limit does not impede its practicability.

Although great efforts were made to create an experimental setting with the brake simulator that would allow us to reliably test emergency braking, the complexity of real driving cannot be entirely simulated under artificial conditions. In particular, the urgency and strength required in a vital emergency could override arthrogenic muscle inhibition.

### *Conclusion*

Although a definite forensic statement is not possible, a simple reaction timer could provide a low‐cost first estimate of BRT for patients and their treating physicians. Nonetheless, until the transferability of the results obtained from a brake simulator or a reaction timer setup to real traffic conditions can be demonstrated, brake simulator measurements will remain the gold standard.
